# Resection of Recurrent Metastatic Osteosarcoma After Bilateral Pulmonary Metastasectomy

**DOI:** 10.1002/ccr3.71559

**Published:** 2025-12-19

**Authors:** Koshi Mobara, Tsuyoshi Uchida, Mamoru Muto, Yuu Tsukahara, Aya Sugimura, Hirochika Matsubara

**Affiliations:** ^1^ Department of Thoracic Surgery Yamanashi University Chuo Yamanashi Japan

**Keywords:** osteosarcoma, pulmonary metastasectomy, pulmonary metastases, recurrence, respiratory function tests, surgical resection

## Abstract

If lung function permits, resectioning recurrent pulmonary metastases can contribute to improved long‐term survival. Additionally, three‐dimensional computed tomography could be useful for lung function assessment.

## 1 Introduction

Osteosarcomas often recur with pulmonary metastases, and effective disease control via resection has been reported [1]. However, repeated lung resection for recurrences requires careful consideration to preserve respiratory function and promote long‐term survival. Herein, we report a case of bilateral multiple pulmonary metastases discovered after surgical resection of osteosarcoma after a 7‐year recurrence‐free period following a previous pulmonary resection.

## 2 Case History/Examination

**FIGURE 1:**
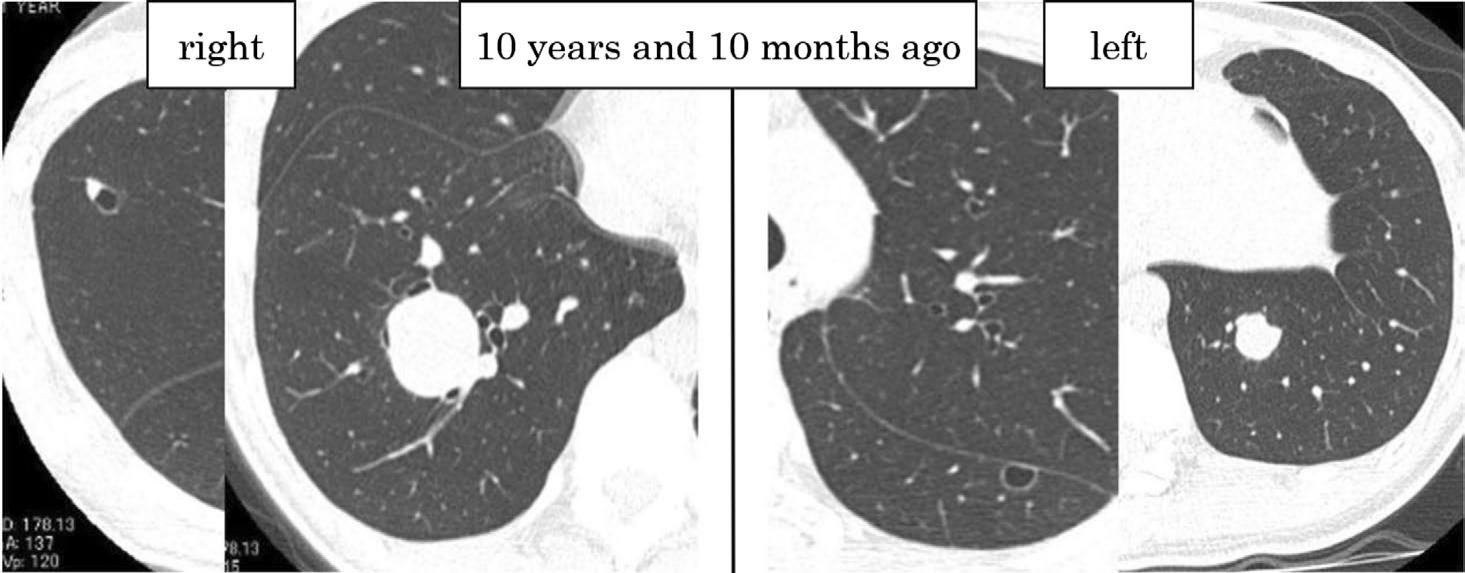
Computed tomography reveals multiple tumors in the right upper, right lower, and left lower lobes after the first metastasectomy.

A 20‐year‐old woman underwent neoadjuvant chemotherapy (one session of intra‐arterial chemotherapy with cisplatin, followed by one course of combination chemotherapy with doxorubicin, cisplatin, ifosfamide, and methotrexate) and joint replacement surgery for osteosarcoma in the right distal femoral diaphysis at 12 years and 7 months prior to the current presentation. The pretreatment diagnosis was high‐grade and T2 according to the Enneking classification. Although there was a possibility of postoperative residual tumor, the response to chemotherapy was equivalent to that of Grade II. She received two additional postoperative courses of the same preoperative combination chemotherapy regimen. Computed tomography (CT) revealed a pulmonary nodule, and the patient underwent wedge resection of the left lower lobe. CT performed 10 years and 10 months before the current presentation showed bilateral pulmonary nodules, after which the patient underwent right lower lobectomy, partial right upper lobe resection, and left lower lobe wedge resection (Figure 1).

## 3 Differential Diagnosis, Investigations, and Treatment

**FIGURE 2:**
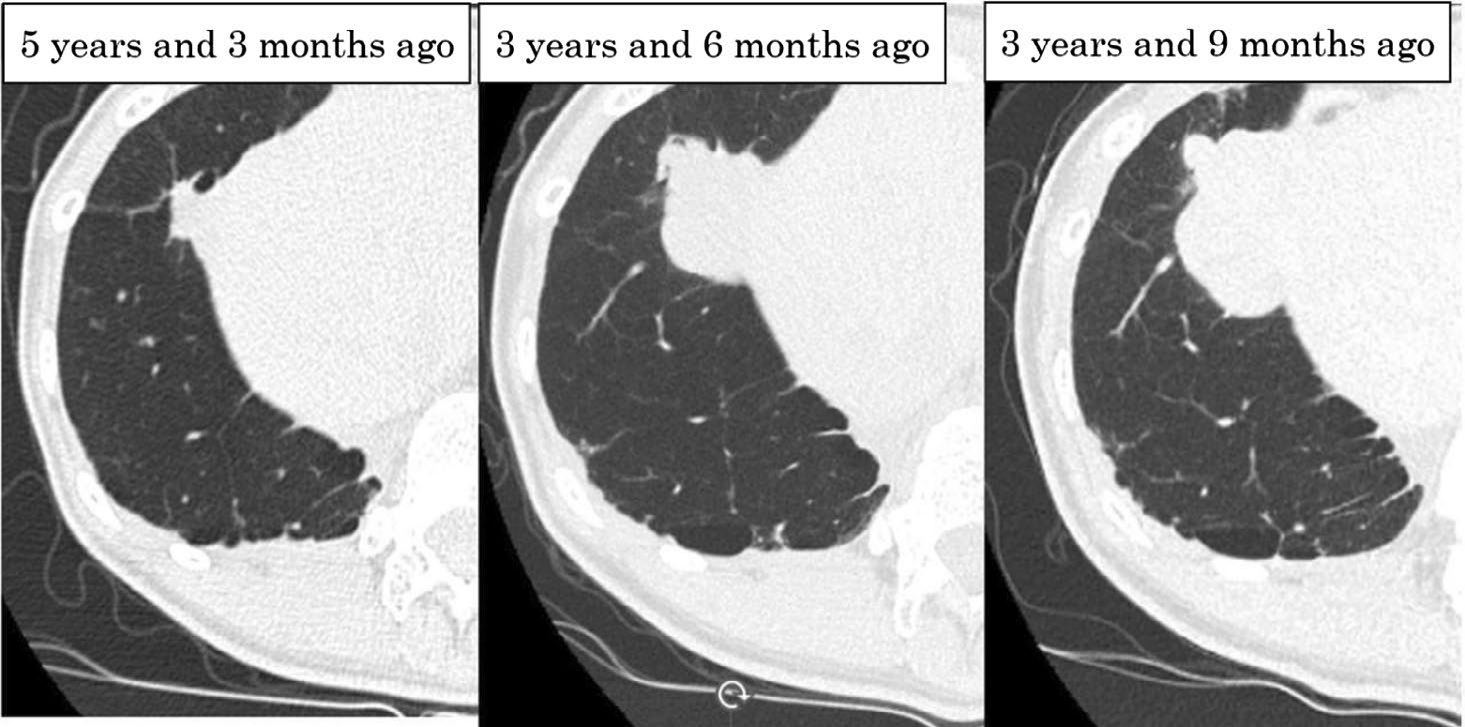
Computed tomography scan showing a nodule in the right middle lobe. The nodule increased in size to 4.5 cm over 18 months.

**FIGURE 3:**
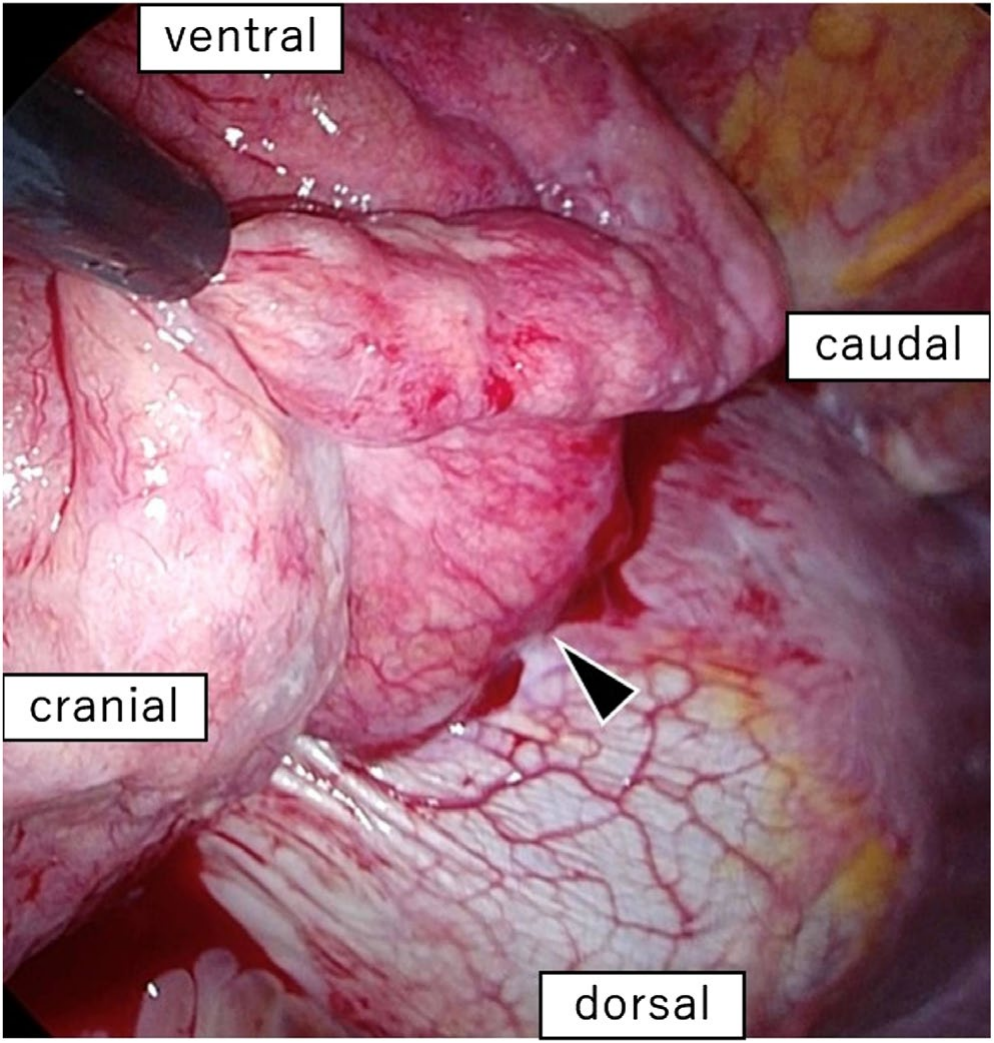
Intraoperative findings. Wedge resection of the right middle lobe and a diaphragm resection were performed using four‐port thoracoscopic surgery. The black arrowhead indicates adhesions between the lungs and the diaphragm. The diaphragm required repair after the wedge resection.

CT revealed another pulmonary nodule in the right middle lobe 5 years and 3 months before presentation. The nodule gradually increased in size for 18 months to 4.5 cm and no other metastases were observed (Figure 2). Respiratory function showed sufficient improvement at 7 years and 9 months after the second pulmonary resection (Table 1); thus, pulmonary resection was repeated. The patient underwent complete thoracoscopic surgery using four ports. Intrathoracic analysis showed that the nodule measured approximately 5 cm and invaded the diaphragm, necessitating resection within the diaphragm. The diaphragm was repaired using Polysorb 2 and 0 sutures (Medtronic, Minneapolis, MN, USA) (Figure 3). The surgery was completed without complications.

**TABLE 1:** Summary of pulmonary function tests measured using spirometry and lung volumes assessed using three‐dimensional computed tomography from the first to third lung resection.

	First resection		Second resection		Third resection	
	Pre	Post	Pre	Post	Pre	Post
FVC (mL)	2540	2280	2690	1980	2420	2080
3D‐CT (mL)	3916.5	4028.5	4031.5	3760.7	3650.2	3699.9
Difference	1376.5	1748.5	1341.5	1780.7	1230.2	1619.9

Abbreviations: 3D‐CT, three‐dimensional computed tomography; FVC, forced vital capacity.

## 4 Outcome and Follow‐Up

**FIGURE 4:**
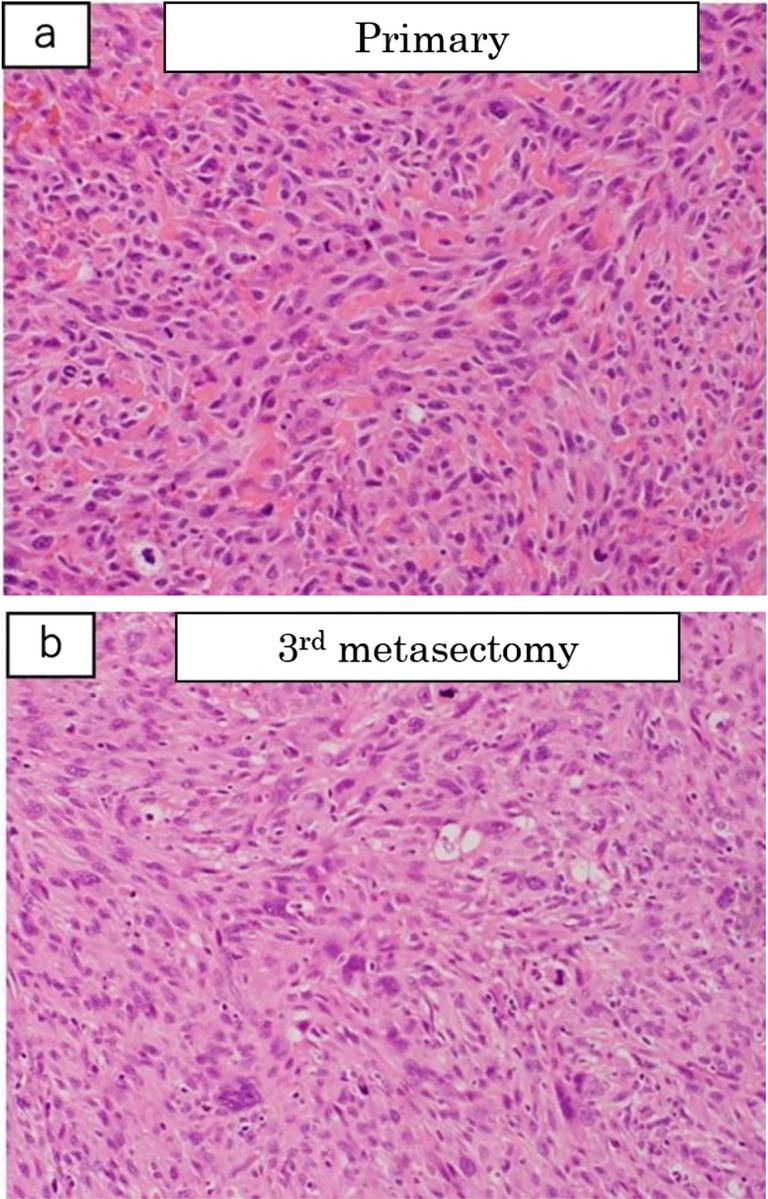
(a) Histopathological features of the primary osteosarcoma. (b) Histology of the lung nodule. The structures are consistent, supporting the diagnosis of metastasis. Pathological examination of the resected specimen indicated the presence of spindle cells similar to those observed in primary osteosarcoma.

Pathological examination of the resected specimen indicated the presence of spindle cells similar to those observed in primary osteosarcoma (Figure 4). The patient did not experience recurrence or dyspnea postoperatively for 2 years and 7 months.

## 5 Discussion

The present case highlights two important aspects. First, resection of pulmonary metastases from osteosarcoma, even if repeated, can improve the prognosis. Several studies have reported the efficacy of resectioning pulmonary metastases from osteosarcoma. Prognostic factors include disease‐free survival (DFS), tumor doubling time, complete resection, and a margin length‐to‐tumor size ratio greater than 0.5 [2, 3, 4]. In the present case, the duration between the primary surgery and the first and second lung metastasectomies was short, whereas that between DFS and the third metastasectomy was long. Additionally, the tumor growth rate was low during the last recurrence period. Therefore, the prognosis was considered favorable.

Second, three‐dimensional (3D) CT volumetry may be useful for evaluating lung function. Iwano et al. [5] and Matsuo et al. [6] found significant correlations between 3D‐CT volumetry of the lungs and spirometry results. Bakker et al. comprehensively discussed the potential challenges in using 3D‐CT volumetry to assess lung function [7].

In our case, the postoperative decrease in lung capacity gradually approached the values indicated by 3D‐CT volumetry over time, with ongoing rehabilitation. This suggests that the values obtained from 3D‐CT volumetry can serve as lung function targets during rehabilitation. Using 3D‐CT volumetry, the goal of improving lung capacity can be achieved through rehabilitation.

## 6 Conclusions

The present case demonstrated that the resection of recurrent pulmonary metastases from osteosarcoma, even if repeated, can improve long‐term survival. The prognosis of patients who undergo pulmonary metastasectomy depends on factors such as DFS, tumor growth rate, and complete resection. In this case, the long disease‐free interval and slow growth rate of the recurrent tumor suggested a favorable prognosis. Additionally, 3D‐CT volumetry showed promise for evaluating post‐resection lung function, enabling targeted rehabilitation goals. This case supports the potential of 3D‐CT volumetry in guiding postoperative management and improving patient outcomes.

## Author Contributions

**Koshi Mobara:** conceptualization. **Tsuyoshi Uchida:** writing – original draft. **Mamoru Muto:** data curation. **Yuu Tsukahara:** writing – review and editing. **Aya Sugimura:** writing – review and editing. **Hirochika Matsubara:** supervision.

## Funding

The authors have nothing to report.

## Ethics Statement

The authors have nothing to report.

## Consent

Written informed consent was obtained from the patient.

## Conflicts of Interest

The authors declare no conflicts of interest.

## Data Availability

Data are available upon reasonable request.
